# Dysregulation of the haem-haemopexin axis is associated with severe malaria in a case–control study of Ugandan children

**DOI:** 10.1186/s12936-015-1028-1

**Published:** 2015-12-21

**Authors:** Robyn E. Elphinstone, Frank Riley, Tian Lin, Sarah Higgins, Aggrey Dhabangi, Charles Musoke, Christine Cserti-Gazdewich, Raymond F. Regan, H. Shaw Warren, Kevin C. Kain

**Affiliations:** Sandra Rotman Centre for Global Health, University Health Network-Toronto General Hospital, Toronto, ON Canada; Department of Laboratory Medicine and Pathobiology, University of Toronto, Toronto, ON Canada; Tropical Disease Unit, Department of Medicine, University of Toronto, Toronto, ON Canada; Infectious Disease Unit, Department of Pediatrics, Massachusetts General Hospital, Boston, MA USA; Makerere University College of Health Sciences, Kampala, Uganda; Laboratory Medicine Program (Transfusion Medicine), University Health Network/University of Toronto, Toronto, ON Canada; Department of Emergency Medicine, Thomas Jefferson University, Philadelphia, PA USA

**Keywords:** Severe malaria, Cerebral malaria, Severe malarial anaemia, Haemopexin, Haptoglobin, Haem, Haemoglobin, Experimental cerebral malaria

## Abstract

**Background:**

Malaria is associated with haemolysis and the release of plasma haem. Plasma haem can cause endothelial injury and organ dysfunction, and is normally scavenged by haemopexin to limit toxicity. It was hypothesized that dysregulation of the haem-haemopexin pathway contributes to severe and fatal malaria infections.

**Methods:**

Plasma levels of haemin (oxidized haem), haemopexin, haptoglobin, and haemoglobin were quantified in a case–control study of Ugandan children with *Plasmodium falciparum* malaria. Levels at presentation were compared in children with uncomplicated malaria (UM; n = 29), severe malarial anaemia (SMA; n = 27) or cerebral malaria (CM; n = 31), and evaluated for utility in predicting fatal (n = 19) vs non-fatal (n = 39) outcomes in severe disease. A causal role for haemopexin was assessed in a pre-clinical model of experimental cerebral malaria (ECM), following disruption of mouse haemopexin gene (*hpx)*. Analysis was done using Kruskall Wallis tests, Mann–Whitney tests, log-rank tests for survival, and repeated measures ANOVA.

**Results:**

In Ugandan children presenting with *P. falciparum* malaria, haemin levels were higher and haemopexin levels were lower in SMA and CM compared to children with UM (haemin, p < 0.01; haemopexin, p < 0.0001). Among all cases of severe malaria, elevated levels of haemin and cell-free haemoglobin at presentation were associated with subsequent mortality (p < 0.05). Compared to ECM-resistant BALB/c mice, susceptible C57BL/6 mice had lower circulating levels of haemopexin (p < 0.01), and targeted deletion of the haemopexin gene, *hpx*, resulted in increased mortality compared to their wild type littermates (p < 0.05).

**Conclusions:**

These data indicate that plasma levels of haemin and haemopexin measured at presentation correlate with malaria severity and levels of haemin and cell-free haemoglobin predict outcome in paediatric severe malaria. Mechanistic studies in the ECM model support a causal role for the haem-haemopexin axis in ECM pathobiology.

**Electronic supplementary material:**

The online version of this article (doi:10.1186/s12936-015-1028-1) contains supplementary material, which is available to authorized users.

## Background

Severe malaria (SM), including severe malarial anaemia (SMA) and cerebral malaria (CM), is associated with high fatality rates [1, 2]. Although the pathogenesis of SM remains unclear, fatal disease is associated with increased parasite burdens, excessive inflammation, endothelial activation, and multi-organ dysfunction [1–3]. During malaria infection there is haemolysis of both parasitized and non-parasitized erythrocytes causing the release of cell-free haemoglobin. Cell-free haemoglobin is readily oxidized to methaemoglobin which releases plasma haem. Plasma haem is toxic to vascular endothelium via a number of established mechanisms, including iron-mediated oxidative stress, increased complement activation [4], induction of pro-inflammatory mediators [5], exocytosis of Weibel-Palade bodies [4, 6], increased leukocyte and platelet adhesion [6], and cell death [7]. Under normal physiological states, cell-free haemoglobin and haem are promptly bound by circulating haptoglobin and haemopexin, respectively, leading to their inactivation via various pathways, including by haem-oxygenase 1 [8]. However, in conditions of intravascular haemolysis these protective pathways may be overwhelmed resulting in oxidative stress, reduced nitric oxide bioavailability, inflammation, endothelial activation, and platelet/fibrin microthrombi that together culminate in vascular dysfunction and multi-organ injury.

Malaria causes haemolysis, however the key components of the haem axis, specifically haemopexin and its role, have not been well studied in SM. This study tests the hypothesis that severe malarial syndromes are associated with higher plasma haem and lower levels of haemopexin and haptoglobin. Using a case–control study the association of the haem axis with severe and fatal malaria in African children was investigated. These investigations were extended to explore a potential mechanistic role for haemopexin using a pre-clinical animal model.

## Methods

### Study participants

This case–control study was nested within a large, observational, prospective study of febrile children (0.5–10.6 years) presenting with smear-confirmed *Plasmodium falciparum* malaria to Mulago Hospital [9]. Mulago Hospital is the district hospital for Kampala, which is located in central Uganda, a region with moderate or meso-endemicity. All study participants were local residents. Children were eligible for enrolment in this study if they met WHO criteria for SMA (Hb <5.0 g/dL) or CM [BCS <3, either before or >6 h after seizures (or anticonvulsant medication), OR >3 seizures witnessed within a 24-hour period; and, absence of hypoglycemia (<2.2 mM), lumbar puncture evidence of bacterial meningitis or intracranial haemorrhage or any known non-malaria-related neurologic abnormalities which may alternatively explain coma] [10], or had uncomplicated malaria (UM) (Hb >7.0 g/dL, platelet count >100,000/uL, with absence of hypoxia, lactic acidosis, respiratory distress, seizures, and coma); and, had a pre-treatment plasma sample collected at presentation and stored frozen (without a freeze–thaw) until analysis. No children had a history of haemoglobinopathy. All of the children in this study were tested for haemoglobin S (HbS) using SickleDex^®^ (Streck Laboratories, Omaha, NB, USA) (except n = 6). Children were excluded from the larger trial if they had severe malnutrition that necessitated transfer to the Nutrition Ward (based on clinical judgement of the attending physician); or if they had a history of HIV/AIDS or a positive HIV ELISA test [9]. Children are routinely screened for HIV as part of their clinical care. Children were tested for HIV using a screening assay, and any positive tests were confirmed with a second ELISA. All children were treated according to Ugandan national malaria guidelines for the treatment of SM and standard operating procedures at Mulago Hospital [9]. This study was approved by the Mulago Hospital Research Ethics Committee, Makerere University, Faculty of Medicine Research Ethics Committee, Uganda National Council for Science & Technology, and the University Health Network. Written informed consent was provided by a parent/guardian prior to enrolment.

Plasma haem is readily oxidized to haemin, therefore, plasma haemin was measured using a colorimetric assay (BioVision, Milpitas, CA, USA). ELISA was used to measure concentrations of cell-free haemoglobin (Bethyl Laboratories, Montgomery, TX, USA), haemopexin and haptoglobin (GenWay Biotech, San Diego, CA, USA). The limits of detection were: haemin 0.5–600 µM; cell-free haemoglobin 22–16,000 µg/mL; haemopexin 63–4000 µg/mL, and haptoglobin 2–2000 µg/mL. A standard curve was run for each assay with manufacturer’s standards, as well as normal human serum to confirm no assay-to-assay variation.

### Experimental cerebral malaria (ECM)

#### Determining endogenous levels of haemopexin in ECM-resistant and -susceptible mice

Wild type C57BL/6 mice (highly susceptible to ECM) and wild type BALB/c mice (moderately resistant to ECM) were purchased from Jackson Laboratories (Sacramento, CA, USA). Female mice were used between eight and 12 weeks of age. *Plasmodium berghei* ANKA was obtained from MR4 (MRA-311) and cryopreserved stocks were passaged through mice prior to infection. Mice were infected intraperitoneally with 10^6^*P. berghei* ANKA-infected erythrocytes. On the day of tissue collection (either day 6 or day 7 post infection, dependent on infection kinetics), heparinized plasma was collected via cardiac puncture.

#### Determining the susceptibility of haemopexin-deficient mice

Haemopexin heterozygous mice were kindly provided by Dr Frank Berger (University of South Carolina), and were originally derived from Tolosano et al. [11]. These mice are on a mixed C57BL/6; 129S6/Sv background and heterozygous mice were bred. In all experiments, littermates, both males and females ages 8–12 weeks, were used. Mice were infected with 10^5^*P. berghei* ANKA-infected erythrocytes injected intraperitoneally. The mice were followed daily for peripheral parasitaemia determined by thin blood smear. Infected mice followed for survival were euthanized when moribund, which included signs of ECM, including limb paralysis, seizures and coma, or a greater than 20 % decrease in body weight. Plasma was collected from infected mice via saphenous vein bleeds or when moribund via cardiac puncture. Animal studies were reviewed and approved by the University Health Network Animal Care Committee. Murine plasma was stored frozen at −80 °C until analysis. ELISAs were used to measure plasma levels of murine haptoglobin and murine haemopexin (Immunology Consultants Laboratory, Portland, OR, USA), and haemin was measured using a colorimetic assay (BioVision, Milpitas, CA, USA). The limits of detection for the murine markers were: haemopexin 125–16,000 ug/mL, haptoglobin 39–5000 ug/mL and haemin 0.2–8.4 uM.

### Statistical analysis

Survival was analysed using the log-rank test; repeated measures ANOVA was used to compare levels of murine haemopexin or haptoglobin over time between genotypes; non-parametric analyses was used to compare plasma levels, either Mann–Whitney tests or Kruskal–Wallis analysis followed by Dunn’s Multiple Comparison tests. All statistical analyses were performed using Graph Pad Prism 6 or IBM SPSS Statistics Version 22.

## Results

### Children with severe malaria

**Fig. 1:**
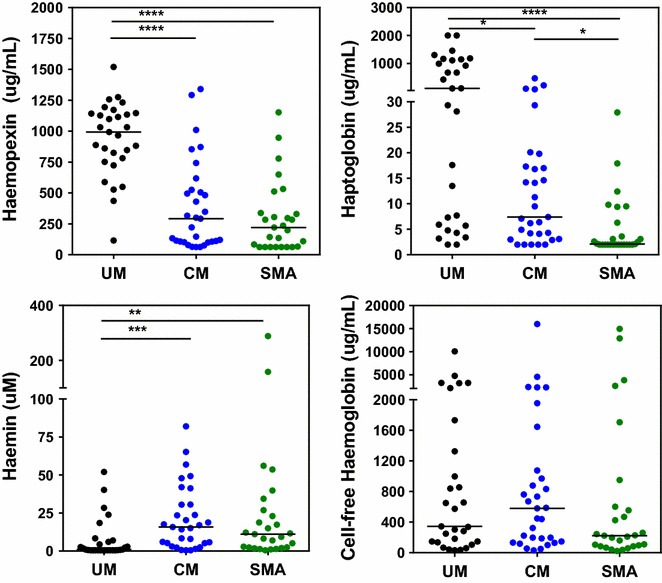
Alterations of the haem axis are associated with disease severity in children with malaria. Plasma samples were collected at presentation. Higher plasma levels of haemin, an oxidized form of haem, and lower levels of haemopexin and haptoglobin were associated with SMA and CM in Ugandan children compared to children with UM. Kruskal–Wallis Test followed by Dunn’s Multiple Comparison Test, *p < 0.05, **p < 0.01, ***p < 0.001, ****p < 0.0001

This study included cases of SM, either CM (n = 31) or SMA (n = 27), and children with UM (n = 29) as controls. Of the children with SM, there were 19 deaths and 39 survivors. None of the children had sickle cell disease (HbSS), and six of the children had sickle cell trait (HbAS) (UM, n = 2; SMA, n = 2; CM, n = 2; including one death). Patient characteristics are shown in Table 1. At presentation children with SMA had 11-fold [median (IQR): 11.1 µM (2.4, 26.9), p < 0.01] and CM had 15-fold higher [15.8 (5.2, 30.6), p < 0.001] median plasma levels of haemin compared to children with UM [1.0 (0.5, 6.5); Fig. 1]. Children with UM had significantly higher levels of haemopexin [993 µg/mL (766, 1144)] than those with SMA [221 (63, 338); p < 0.0001] or CM [292 (106, 525); p < 0.0001; Fig. 1]. Similarly, haptoglobin levels were significantly higher in patients with UM [109 µg/mL (6, 1130)] compared to those with SMA (2 [2, 6]; p < 0.0001) or CM (7 [3, 17]; p < 0.05). Haptoglobin levels were significantly lower in children with SMA compared to CM (p < 0.05; Fig. 1).

**Table 1:** Descriptive characteristics of the patient population

					Combined SM (All children with SMA or CM)		
Characteristic	UM (n = 29)	CM (n = 31)	SMA (n = 27)	P value	Survivors (n = 39)	Deaths (n = 19)	P value
Gender (% female)	48.3	58.1	48.1	0.679	48.7	63.2	0.301
Age (years)	3.2 (2.1, 7.4)^***^	3.1 (1.5, 4.4)^##^	1.3 (1.0, 2.2)^##,***^	0.000	1.9 (1.0, 4.0)	2.1 (1.3, 3.4)	0.673
Days reported ill prior to presentation	3 (2, 4)^*^	3 (2, 4)^##^	4 (3, 5)^*,##^	0.004	3 (3, 4)	3 (2, 5)	0.496
Parasitaemia (parasites/uL)	6.0 × 10^4^ (9.9 ×10^3^, 1.4 × 10^5^)	8.2 × 10^4^ (1.8 × 10^4^, 2.8 × 10^5^)	3.2 × 10^4^ (5.3 × 10^3^, 1.4 × 10^5^)	0.090	3.7 × 10^4 ^ (6.2 × 10^3^, 1.4 × 10^5^)	1.6 × 10^5^ (1.8 × 10^4^, 4.4 × 10^5^)	0.106
Red blood cell haemoglobin (g/dL)	10.0 (9.2, 11.3)^***,$$^	7.1 (5.8, 9.3)^###,$$^	3.8 (3.2, 4.6)^###,***^	0.000	4.7 (3.5, 7.1)	5.4 (4.6, 8.3)	0.134
Platelet count (x10^9^/L)	162 (104, 258)^$$$^	67 (43, 128)^$$$^	126 (86, 180)	0.001	95 (50, 174)	73 (41, 128)	0.230
Weight (kg)	14 (11, 22)^***^	13 (10, 15)^##^	10 (7.5, 11)^##,***^	0.000	10 (8, 14)	11 (10, 13)	0.269
Fatal cases (count)	0	13	6		0	19	

Continuous variables analysed by Mann–Whitney, or Kruskall Wallis with Dunn’s multiple comparisons; categorical variable analysed by Chi square analysis

Median (IQR), uncomplicated malaria (UM), cerebral malaria (CM), severe malarial anaemia (SMA), severe malaria (SM)

Dunn’s multiple comparison tests: UM vs SMA * p < 0.05, *** p < 0.001; UM vs CM ^$$^ p < 0.01, ^$$$^ p < 0.001; CM vs SMA ^#^ p < 0.05, ^##^ p < 0.01, ^###^ p < 0.001

**Fig. 2:**
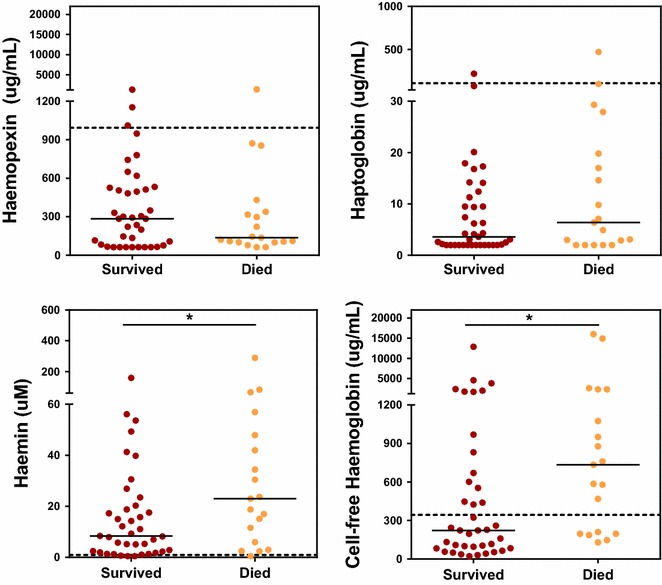
Plasma levels of haemin and cell-free haemoglobin are associated with disease outcome in severe malaria. Children who died of SM, either SMA or CM, had higher plasma levels of haemin and cell-free haemoglobin at presentation compared to children who survived SM. Levels of haemopexin and haptoglobin were lower than those observed in children with UM but were not significantly different between those who survived SM and those who did not. The *dotted line*s indicate the median levels observed in children with UM. Mann–Whitney test, *p < 0.05

Since both children with CM or SMA showed similar evidence of dysregulation of the haem axis, the association of haemin and cell-free haemoglobin levels with outcome was then examined. Among children with SM (CM and SMA cases combined) plasma levels of both haemin [23.0 µM (6, 47.9) vs 8.4 (2.3, 20.30), p = 0.03] and cell-free haemoglobin [734.0 µg/mL (196.0, 2255) vs 222.0 (84.0, 669), p = 0.01] were significantly higher at presentation in those who subsequently went on to die of their infection compared to children who survived (Fig. 2). Sub-group analysis of children with either CM or SMA showed similar trends with respect to levels of haemin and cell-free haemoglobin and an increased risk of fatal outcome but these did not reach statistical significance (Additional file 1: Figure S1).

Excluding the children with the protective sickle cell trait (HbAS) from the analysis did not alter the associations of the haem axis markers. In summary, these data are consistent with the hypothesis that severe malarial syndromes are associated with changes in the haem axis.

### Experimental cerebral malaria

**Fig. 3:**
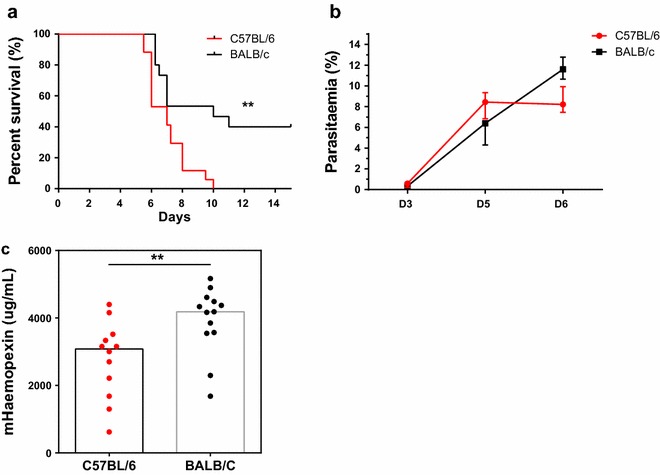
Endogenous levels of haemopexin are associated with susceptibility to experimental cerebral malaria. **a** BALB/c mice are more resistant to ECM than C57BL/6 mice as demonstrated by improved survival (pooled data from two biological replicates; n = 15–17 per group; log rank test, **p < 0.01). Both strains of mice had similar levels of **b** parasitaemia throughout the course of infection (representative data from a single experiment; n = 7-8/group); **c** BALB/c mice had higher levels of plasma mouse (m) haemopexin compared to the more susceptible C57BL/6 mice when moribund (pooled data from two biological replicates; n = 12–13/group). *mHpx* Mann–Whitney test; **p < 0.01

**Fig. 4:**
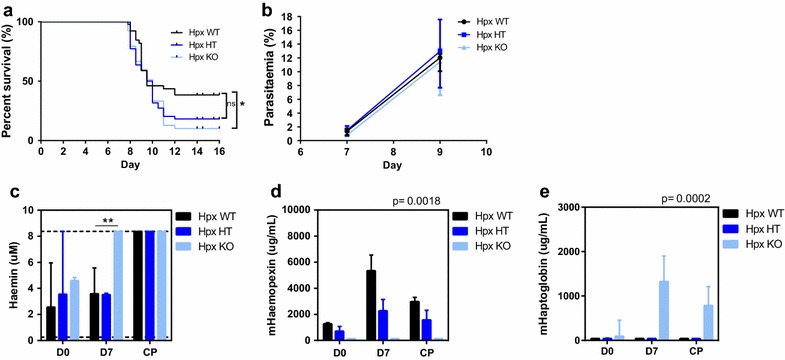
Haemopexin-deficient mice are more susceptible to experimental cerebral malaria. **a** Both haemopexin knockout (Hpx KO) and haemopexin heterozygous (Hpx HT) mice have decreased survival during ECM than their wild type (Hpx WT) littermates (pooled data from four biological replicates; n = 39–44/group; log rank test, *p < 0.05). All three genotypes had similar levels of **b** parasitaemia (representative data from a single experiment; n = 7–12/group). The Hpx KO animals had significantly higher levels of **c** plasma haemin on day 7 post infection, Mann–Whitney, **p < 0.01. The *dotted line* indicates upper limit of detection for the assay (8.4 uM). The plasma levels of **d** haemopexin, and **e** haptoglobin throughout the course of infection (n = 4/group); two-way ANOVA, **d** p = 0.0018, **e** p = 0.0002. Samples were collected on day 0 and day 7 post infection, then at cardiac puncture (CP) when mice were moribund

BALB/c mice infected with *P. berghei* ANKA had significantly improved survival compared to the C57BL/6 mice (40 % survival vs 0 %, p = 0.003) with similar levels of parasitaemia during the course of infection (Fig. 3a, b). When the susceptible mice were exhibiting signs of ECM (day 6 or day 7 post infection), the plasma levels of haemopexin were significantly higher in BALB/c mice compared to the more susceptible C57BL/6 mice [4185 ug/mL (3557, 4550) vs 3080 ug/mL (1814, 3472), p = 0.005; Fig. 3d]. Furthermore, mice with targeted disruption of the *hpx* gene had significantly decreased survival compared to their wild type littermates (10 vs 38 % survival, p = 0.022; Fig. 4a). This was associated with significantly higher levels of plasma haemin at day 7 post infection in the haemopexin knockout animals compared to the wild type animals [>8.4 uM (7.3, 8.4) vs 3.6 (3.2, 5.6), p = 0.005; Fig. 4c]. The haemopexin heterozygous mice had approximately half the circulating levels of haemopexin than their wild-type counterparts throughout the course of infection (Fig. 4d) but responded more similarly to the haemopexin-null mice. There was a trend towards a difference in survival between the haemopexin heterozygous mice compared to their wild-type littermates but it did not reach statistical significance (18 vs 38 % survival, p = 0.068; Fig. 4a). There were no differences observed in peripheral parasitaemia (Fig. 4b) or weight loss between the genotypes. Haemopexin knockout animals and their wild-type counterparts succumb to ECM during the ECM window (days 6–10); these mice begin to die roughly 2 days later than the C57BL/6 mice due to a lower inoculum (10^5^ vs 10^6^ parasitized erythrocytes, respectively). The mice that survived this period succumbed to anaemia/hyperparasitaemia and/or had to be euthanized due to weight loss (>20 %).

In response to acute haemolysis, haemopexin-deficient mice increase their plasma levels of haptoglobin to a greater degree than their heterozygous or wild-type littermates throughout the course of infection (p = 0.0002; Fig. 4e). Mice deficient in haemopexin displayed higher median levels of haptoglobin at baseline prior to infection (89 ug/mL compared to undetectable) compared to their wild-type counterparts. Following infection, haptoglobin levels in the haemopexin-null mice reached a peak at day 7 post infection with a median of 1324 ug/mL while the levels in both the haemopexin wild type and haemopexin heterozygous mice remained undetectable. When the mice were moribund, the haptoglobin levels in the haemopexin-deficient mice had dropped to a median of 783 ug/mL, while the other genotypes continued to have undetectable levels of haptoglobin. Together, these data are consistent with the hypothesis that haemopexin is playing a mechanistic role during ECM.

## Discussion

SM is a complex, multi-organ syndrome that is poorly understood. This study provides evidence that increased generation of plasma haem, combined with lower levels of haemopexin that normally binds and clears haem, are associated with both SMA and CM in Ugandan children. Furthermore, decreased or depleted levels of haemopexin, and increased plasma haem, are associated with a worse outcome in a pre-clinical model of CM. These observations support the hypothesis that haem-induced pathology, exacerbated by decreased levels of haemopexin, may be a pathway of injury contributing to disease severity and death in malaria infections.

The observed median plasma levels of haemin in children with CM and SMA were ten-fold or higher than that observed in UM [12], and equivalent to those reported in haemolytic disorders such as sickle cell disease [4], where haem-induced vasculopathy is a frequent cause of organ dysfunction and mortality [8]. Although there were no differences in cell-free haemoglobin levels between groups, given the 11–15-fold higher levels of plasma haemin observed in children with SMA and CM and that cell-free haemoglobin generates haem, it is likely that there were also differences in levels of plasma haemoglobin during the course of infection, but the timing of sample collection did not capture these events.

Previously it has been shown that plasma levels of haem are higher on day 6 post infection in the ECM-susceptible C57BL/6 mice compared to ECM-resistant BALB/c mice [13]. Injections of haemin into resistant BALB/c mice infected with *P. berghei* ANKA resulted in increased fatalities compared to controls (100 vs 0 %) [13, 14], suggesting that haem may play an important role in mediating, or enhancing, disease severity. While a role for haem has previously been implicated in ECM, it had yet to be confirmed in informative human populations. Recently, Dalko et al. showed that adults with SM in India had elevated levels of plasma haem compared to those with mild malaria, which were both higher than levels measured in endemic controls [15]. These findings were extended in this study to show an association between increased haem and disease severity in African children with SM compared to those with UM.

It is hypothesized that haem is mediating adverse outcomes during malaria infection by inducing endothelial dysfunction and microvascular injury. Haem is implicated, directly or indirectly, as a key mediator of endothelial dysfunction by a variety of pathways. Plasma haem is a major source of reactive oxygen species (ROS) and subsequent pro-oxidant stress on endothelium [8]. Haem also induces activation of the endothelium by: inducing Weibel-Palade body exocytosis and the release of their contents, including the release of von Willebrand factor (vWF), p-selectin [4, 6], and Ang-2, and increasing expression of endothelial adhesion markers that bind both inflammatory cells and parasitized erythrocytes [6]. Haem is also a pro-inflammatory molecule that can stimulate complement activation [4] and the release of pro-inflammatory cytokines from monocytes/macrophages [16]. Haem-induced endothelial injury may be further exacerbated by decreases in bioavailable nitric oxide. Cell-free haemoglobin in addition to generating plasma haem, is a potent scavenger of nitric oxide, further amplifying endothelial dysfunction [8, 17]. Many of these pathways induced by haem are also implicated in the pathogenesis of SM [3, 17–23]. Further prospective studies are required to define which of the above pathways are induced by haem in the context of SM.

The toxic effects of cell-free haemoglobin and haem are normally mitigated by high affinity-binding to haptoglobin and haemopexin, respectively, followed by transport and degradation. Haem is degraded by haem oxygenase-1 resulting in the generation of biliverdin and carbon monoxide which have anti-inflammatory and endothelial stabilizing effects [8]. Therefore, the excess generation of haemin observed in children with CM and SMA appears to be further complicated by a relative deficiency in circulating haemopexin. Previous studies have primarily focused on levels of haptoglobin [12, 24–26] or haemopexin [24–26] in UM. However, it was recently shown that adults with SM in India had decreased levels of haemopexin compared to those with mild malaria [15]. Similarly, in this study it was observed that children with SMA had significantly lower levels of both haemopexin and haptoglobin, in agreement with a previous report [25]. This study extends these previous findings and reports that African children with CM also have lower levels of both haemopexin and haptoglobin, suggesting a common pathway of endothelial injury in both CM and SMA, and in both adults and children with severe disease. Despite the markedly low levels of these protective proteins in children with CM or SMA, there were no observed differences in either haemopexin or haptoglobin levels between children who survived or died of the infection. These data suggest that these proteins are already depleted during severe infection. Whether these relative deficiencies are due to consumption or due to a decreased capacity to synthesize these proteins in response to haemolysis is unknown. Nonetheless, these low levels would be expected to further compromise clearance and degradation of haem and haemoglobin, and contribute to worse clinical outcomes, as was illustrated by the observed higher plasma levels of haemin and cell-free haemoglobin in children who subsequently died of malaria.

Due to the associations observed in children with CM or SMA, a potential mechanistic role for haemopexin was investigated using a pre-clinical model of ECM. As with all animal models, there are inherent limitations. However, there is evidence that this model shares several features, but not all, with human malaria [27–32]. Pre-clinical models enable investigation of mechanistic questions that are difficult to safely or ethically address in human populations and can provide direct genetic evidence that the haem axis is causally involved in severe and fatal infection.

Similar to this patient population, mice that were more susceptible to ECM had significantly lower haemopexin levels compared to more resistant mice, despite comparable parasite burdens. Furthermore, mice with targeted deletion of the *hpx* gene, and no measurable circulating haemopexin, were more susceptible to ECM than their wild-type littermates suggesting that haemopexin is playing a mechanistic role during malaria infection. The increased susceptibility in the Hpx KO animals was associated with increased levels of plasma haemin prior to the onset of ECM, further implicating a role for plasma haem.

A dose response to ECM was predicted due to the gene dosing effect observed in these mice; however, both the haemopexin-null and heterozygous mice responded similarly to ECM. The haemopexin-null mice appear to compensate for their lack of haemopexin by increasing the production of haptoglobin. The haemopexin-null mice have higher baseline levels of haptoglobin compared to both the wild type and heterozygous mice. When infected with *P. berghei* ANKA, haemopexin-deficient mice further upregulate circulating haptoglobin to at least 1300 times the levels seen in either the wild type or heterozygous mice. This compensation in a protein upstream of haemopexin in the haemolytic pathway is hypothesized to mitigate the severity of the observed phenotype. The haemopexin heterozygous mice may more accurately reflect the impact of haemopexin deficiency in ECM due to their inability to compensate with increased levels of haptoglobin to the levels observed in haemopexin-null mice. It would be of interest to further explore the phenotype of a double haemopexin, haptoglobin knockout mouse in the ECM model.

Despite the marked increase in haptoglobin, haemopexin-null mice are still more susceptible than their wild-type counterparts. This is likely attributable to malaria-induced generation of plasma haemoglobin and haem that overwhelms both haptoglobin and/or haemopexin pathways, as demonstrated by significantly higher levels of plasma haem prior to the onset of ECM in the KO animals. Alternatively, haemopexin may provide additional protection independent of its ability to clear haem from the circulation. Haemopexin has been shown to exert haem-independent, anti-inflammatory effects by decreasing the release of pro-inflammatory cytokines from lipopolysaccharide (LPS) stimulated macrophages [33], and decreased differentiation of TH17 cells in a model of experimental autoimmune encephalitis [34].

Collectively, the above observations suggest that malaria-induced haem, and corresponding haemopexin deficiency may represent a component of disease pathogenesis in which haem amplifies complement activation, pro-coagulant activity and endothelial injury, culminating in microangiopathy, multi-organ dysfunction and adverse clinical outcomes. The recent observation that both cases of CM and SMA are associated with parasitized erythrocytes that bind to endothelial protein C receptor (EPCR) lends further support to this hypothesis [35]. Binding to EPCR may disrupt the anti-inflammatory and endothelial barrier effects of activated protein C (APC), resulting in enhanced pro-coagulant activity, complement activation and endothelial injury. Of note, APC mediates its endothelial cytoprotective and barrier protective activities via the Ang-Tie2 pathway [36], which is dysregulated in SM [37]. Moreover, since parasitized erythrocyte sequestration focuses parasite burdens and erythrocyte haemolysis directly onto the microvasculature of vital organs, this would be expected to result in higher local haem concentrations in the cerebral microvascular bed, than those observed in the peripheral circulation.

Similar to atypical haemolytic uremic syndrome (aHUS) associated with infection, where haem-induced complement activation is a proposed common pathway of microvascular injury and organ failure, severe disease does not occur in all infected individuals, but rather only in those with genetic or acquired defects in complement regulation [4]. This suggests that similar or related susceptibility determinants, including defects in haemopexin expression or haem metabolism, may contribute to the onset and outcome of severe malarial syndromes.

## Conclusions

These data support the hypothesis that elevated plasma haem contributes to the pathobiology of severe and fatal malaria. Future studies will need to confirm these findings and investigate other potential interactions (e.g., Ang-Tie2 axis) contributing to haem axis dysfunction in children with severe malarial syndromes. The identification of plasma haem as a potential mediator of disease pathogenesis suggests potential therapeutic interventions, including the administration of exogenous haemopexin which has been shown to confer protection to haem-induced endothelial injury in models of chronic haemolysis [8], and in models of sepsis [7].

## Additional file

10.1186/s12936-015-1028-1 Alterations in the haem axis in children with (A) severe malarial anaemia or (B) cerebral malaria based on disease outcome. There was a trend towards higher plasma levels of haemin and cell-free haemoglobin at presentation in children who died of the infection compared to those who survived; however, these associations are likely underpowered to reach statistical significance (haemin: SMA p = 0.14, CM p = 0.14; haemoglobin: SMA p = 0.06, CM p = 0.25). There was no observable difference between levels of haemopexin and haptoglobin and disease outcome in children with either SMA or CM. The dotted lines indicate the median levels observed in children with UM. Mann–Whitney test.

## Abbreviations

CM: cerebral malaria
SMA: severe malarial anaemia
SM: severe malaria
UM: uncomplicated malaria
ECM: experimental cerebral malaria
ROS: reactive oxygen species
EPCR: endothelial protein C receptor
APC: activated protein C
aHUS: atypical haemolytic uremic syndrome
