# Supplementary figures and images for: Dysregulation of the haem-haemopexin axis is associated with severe malaria in a case–control study of Ugandan children
Source: Malar J. 2015 Dec 21;14:511. doi: 10.1186/s12936-015-1028-1 (PMC4687388; doi:10.1186/s12936-015-1028-1)

**A)**

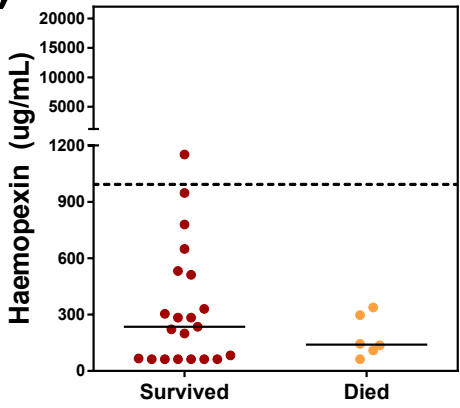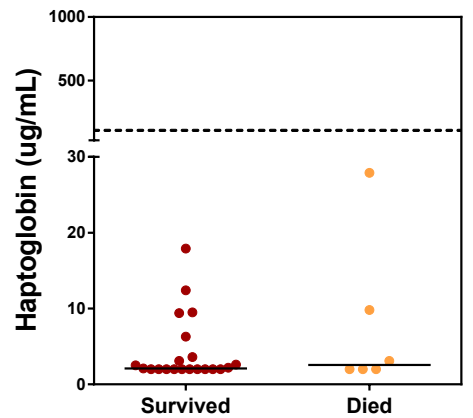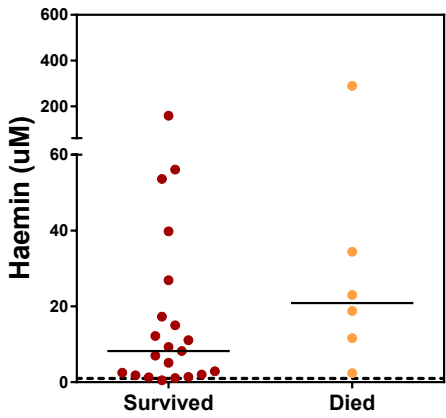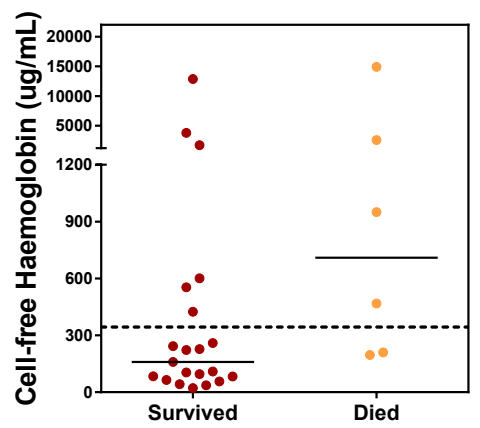

**B)**

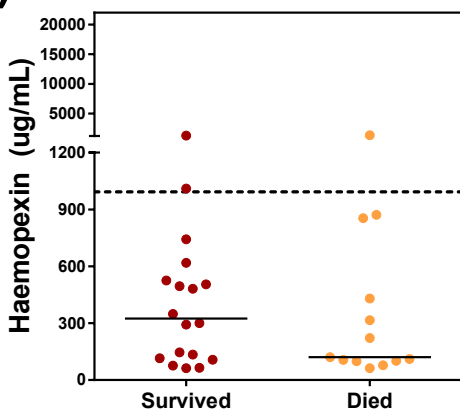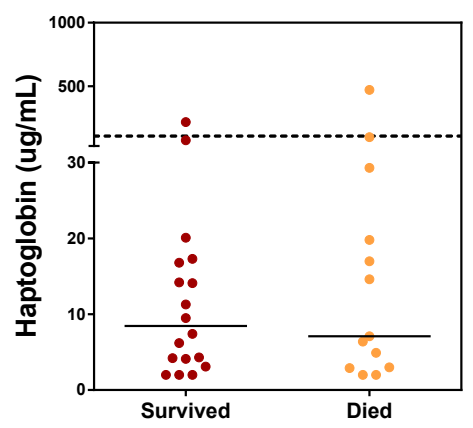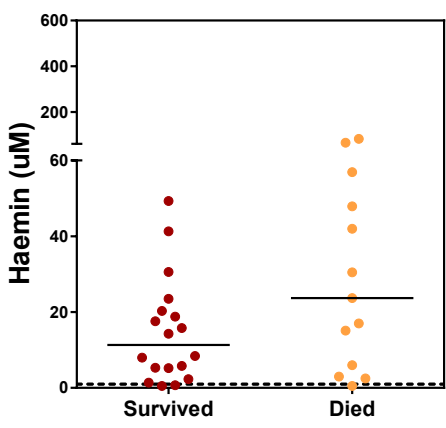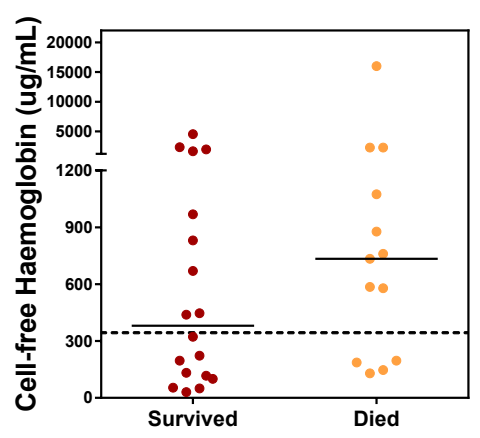

Supplement: Supplementary file 1 — 10.1186/s12936-015-1028-1 Alterations in the haem axis in children with (A) severe malarial anaemia or (B) cerebral malaria based on disease outcome. There was a trend towards higher plasma levels of haemin and cell-free haemoglobin at presentation in children who died of the infection compared to those who survived; however, these associations are likely underpowered to reach statistical significance (haemin: SMA p = 0.14, CM p = 0.14; haemoglobin: SMA p = 0.06, CM p = 0.25). There was no observable difference between levels of haemopexin and haptoglobin and disease outcome in children with either SMA or CM. The dotted lines indicate the median levels observed in children with UM. Mann–Whitney test. [file 12936_2015_1028_MOESM1_ESM.pdf]
